# Pan-Sigma Receptor Modulator RC-106 Induces Terminal Unfolded Protein Response In *In Vitro* Pancreatic Cancer Model

**DOI:** 10.3390/ijms21239012

**Published:** 2020-11-27

**Authors:** Michela Cortesi, Alice Zamagni, Sara Pignatta, Michele Zanoni, Chiara Arienti, Daniela Rossi, Simona Collina, Anna Tesei

**Affiliations:** 1 Biosciences Laboratory, Istituto Scientifico Romagnolo per lo Studio e la Cura dei Tumori (IRST) IRCCS, 47014 Meldola, Italy; a.zamagni86@gmail.com (A.Z.); sara.pignatta@irst.emr.it (S.P.); michele.zanoni@irst.emr.it (M.Z.); chiara.arienti@irst.emr.it (C.A.); 2 Department of Drug Sciences, Medicinal Chemistry and Pharmaceutical Technology Section, University of Pavia, 27100 Pavia, Italy; daniela.rossi@unipv.it (D.R.); simona.collina@unipv.it (S.C.)

**Keywords:** sigma receptors, UPR, pancreatic cancer, ER stress, ROS, gatekeepers

## Abstract

Pancreatic cancer (PC) remains one of the most lethal cancers worldwide. Sigma receptors (SRs) have been proposed as cancer therapeutic targets. Their main localization suggests they play a potential role in ER stress and in the triggering of the unfolded protein response (UPR). Here, we investigated the mechanisms of action of **RC-106**, a novel pan-SR modulator, to characterize therapeutically exploitable role of SRs in tumors. Two PC cell lines were used in all the experiments. Terminal UPR activation was evaluated by quantifying BiP, ATF4 and CHOP by Real-Time qRT-PCR, Western Blot, immunofluorescence and confocal microscopy. Cell death was studied by flow cytometry. Post-transcriptional gene silencing was performed to study the interactions between SRs and UPR key proteins. **RC-106** activated ER stress sensors in a dose- and time-dependent manner. It also induced ROS production accordingly with ATF4 upregulation at the same time reducing cell viability of both cell lines tested. Moreover, **RC-106** exerted its effect through the induction of the terminal UPR, as shown by the activation of some of the main transducers of this pathway. Post-transcriptional silencing studies confirmed the connection between SRs and these key proteins. Overall, our data highlighted a key role of SRs in the activation of the terminal UPR pathway, thus indicating pan-SR ligands as candidates for targeting the UPR in pancreatic cancer.

## 1. Introduction

Pancreatic cancer (PC) is expected to be the second leading cause of cancer-related death by 2030 [1]. Exocrine cancers comprise 95% of all PCs, among which pancreatic ductal adenocarcinoma (PDAC) is the most diffuse histological variant, accounting for 85% of all cases [2,3]. Gemcitabine, capecitabine and 5-fluorouracil (5-FU) still represent the pillar of treatment for non-resectable and borderline-resectable PC [4,5]. Several trials are currently ongoing to identify potentially useful new drugs in PC, nevertheless targeted therapies play a limited role in PDAC management, resulting in a dismal long-term outcome for patients and justifying continued efforts to find novel therapeutic options [6,7,8].

Literature data have shown that malignant cells take advantage of unfolded protein response (UPR) pathways to adapt to an unfavorable microenvironment and to cope with the high rate of misfolded and unfolded proteins deriving from increased metabolic need [9]. Nonetheless, under conditions of intense/persistent endoplasmic reticulum (ER) stress, UPR sensors, inositol-requiring enzyme 1 (IRE1α), PKR-like ER kinase (PERK) and activating factor 6 (ATF6), are capable of switching this pro-survival pathway into a cell death signal mediated by the activation of the so-called “terminal UPR” through the upregulation of activating transcription factor 4 (ATF4) and CCAAT/enhancer-binding protein-homologous protein (CHOP), considered hallmarks of ER-stress induced apoptosis [10,11].

One of the mechanisms known to regulate UPR activation and ER function is ROS generation, which commonly occurs in several diseases, including cancer [12,13]. In particular, ROS generation increases when stressed cells attempt to restore the altered protein folding process. Consequently, oxidative stress contributes to ER stress which, in turn, induces UPR [14]. UPR may also be activated by the alteration, in structure and/or function, of ER chaperones, leading to protein folding impairment [15,16]. In this scenario, the communication between the ER and the mitochondria may guide the final pro-survival or pro-apoptotic outcome of this pathway activation. To fulfill these functions, a large number of chaperones reside in mitochondria-associated membranes (MAMs), among which is the widely studied sigma 1 receptor (S1R) [17,18].

Sigma receptors (S1R and S2R) have been proposed as therapeutic targets for several diseases, among which neurodegenerative disorders, cancer and, recently, COVID-19 [19,20,21,22,23,24,25,26,27]. Interestingly, both receptors are highly expressed in some cancers, including PC, and their ligands are therefore of great interest in the field of pharmacology for the treatment and diagnosis of cancer [28,29,30]. This behavior, the lack of known endogenous ligands and the affinity for a large number of small molecules, has led to SRs, in particular S1R, being considered as ligand-activated chaperones rather than receptors [31,32]. On the basis of this hypothesis, the main functions of S1R appear to be the modulation of Ca^2+^ release through inositol triphosphate receptor type 3 (IP3R) stabilization and the formation of a complex with 78-kDa glucose-regulated protein (GRP78), an ER stress sensor that plays a central role in protein folding [33]. The identity of S2R has been controversial for several years and only recently it has been identified as TMEM97, a transmembrane protein known to reside in the ER and lysosomes with the function of binding cholesterol and regulating the Niemann-Pick C1-Like 1 (NPC1L1) protein [34,35]. Despite the lack of information available on this receptor, its pharmacological profile is similar to that of S1R, probably because of its homology with enzymes sharing the same function: yeast C8-C7 sterol isomerase ERG2p (S1R) and mammalian C8-C7 sterol isomerase emopamil binding protein EBP (TMEM97/S2R) [36]. In agreement with the S1R chaperone model and with the pharmacological properties of S1R and S2R, we recently hypothesized their potential role as ER stress gatekeepers, thus supporting their involvement in the triggering of the terminal unfolded protein response [37]. Our research team has been active in this area for several years, studying SRs from a medicinal chemistry standpoint and also as potential anti-cancer therapeutic targets [38,39,40,41,42,43,44]. In this scenario, we recently identified **RC-106** characterized by a pan-SR modulatory activity, in vitro antiproliferative properties toward cancer cell lines, including PC, and a good pharmacokinetic profile. Moreover, given that tissue distribution in target organs is at the core of the development process because of its direct impact on drug activity, we studied the pharmacokinetic profile of the modulator and evaluated its distribution in the pancreas. Our results showed that **RC-106** was 25-fold more concentrated in the pancreas than in plasma, reaching a concentration higher than that required to be effective in all the in vitro experiments [45]. In light of the above considerations, we used **RC-106** as a drug model to investigate the therapeutic role of SRs in activating the terminal UPR, mediated by ER stress induction.

## 2. Results

### 2.1. **RC-106** Induces Substantial ER Stress and Triggers Terminal UPR

**Figure 1:**
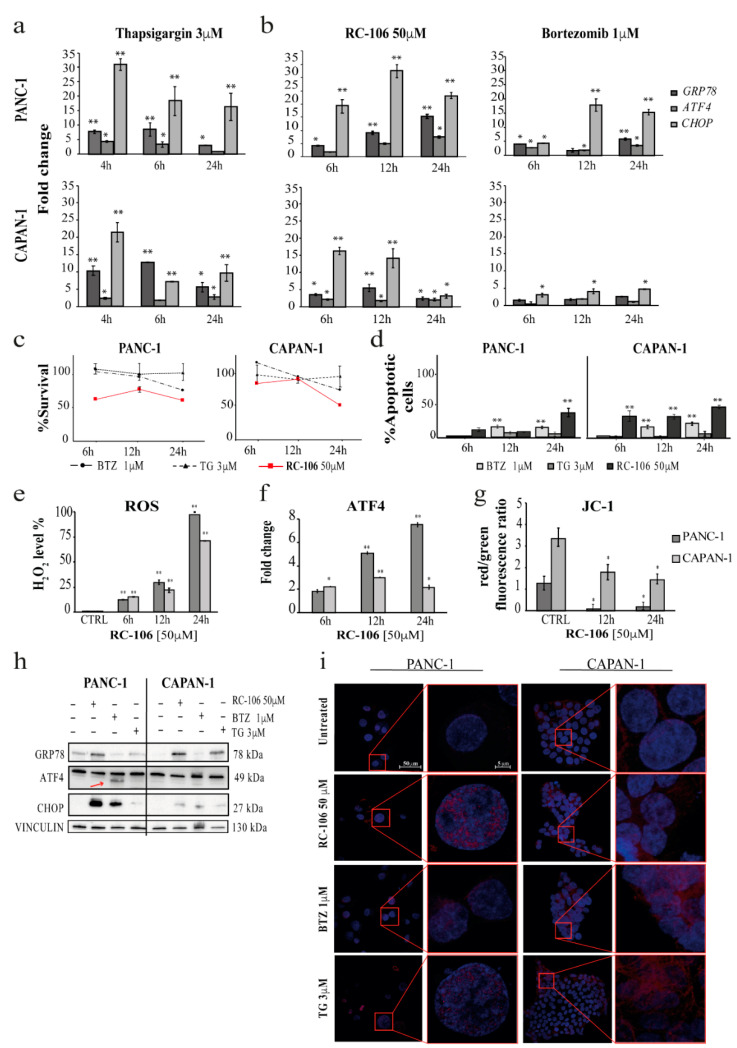
mRNA and protein analysis of GRP78, ATF4 and CHOP expression in Panc-1 and Capan-1 cell lines. (**a**) GRP78, ATF4 and CHOP mRNA upregulation after treatment with TG (3 µM) for 4, 6 and 24 h. Analyses were performed by Real-Time RT qPCR. RNA expression was normalized to GAPDH and HPRT-1. mRNA expression was relative to the corresponding untreated control at each time-point tested (Fold change = 1). Values are the mean ± SD of three independent experiments (* *p* ≤ 0.05, ** *p* ≤ 0.01 vs. CTRL). (**b**) GRP78, ATF4 and CHOP mRNA upregulation after treatment with **RC-106** (50 μM) and BTZ (1 μM) for 6, 12 and 24 h. Analyses were performed by Real-Time RT qPCR. RNA expression was normalized to GAPDH and HPRT-1. mRNA expression was relative to the corresponding untreated control at each time-point tested (Fold change = 1). Values are the mean ± SD of three independent experiments (* *p* ≤ 0.05, ** *p* ≤ 0.01 vs. CTRL). (**c**) Panc-1 and Capan-1 cell survival after treatment with TG (3 µM), BTZ (1 μM) and **RC-106** (50 μM) for 6, 12 and 24 h. MTS assay was used to determine cell viability. Values are the mean ± SD of three independent experiments. (**d**) Apoptosis was analyzed by TUNEL assay. Panc-1 and Capan-1 cells were treated with BTZ (1 μM) and TG (3 μM) and **RC-106** (50 μM) for 6, 12 and 24 h. Values are the mean ± SD of two independent experiments (* *p* < 0.05; ** *p* ≤ 0.01 vs. CTRL). (**e**) ROS (H_2_O_2_) levels measured in Panc-1 and Capan-1 cells untreated or treated with **RC-106** 50 μM for 6, 12 and 24 h. Data are normalized for protein concentration and expressed as percentage changes in mean ROS values with respect to those obtained in untreated cells (** *p* ≤ 0.01 vs. CTRL). (**f**) ATF4 mRNA upregulation after treatment with **RC-106** 50 µM for 6, 12 and 24 h (* *p* ≤ 0.05, ** *p* ≤ 0.01 vs. CTRL). (**g**) Cytofluorimetric analysis of mitochondria depolarization by JC-1 assay. Cells were exposed to **RC-106** 50 μM for 12 and 24 h, and depolarization was expressed as red/green fluorescence ratio. (**h**) GRP78, ATF4 and CHOP protein analysis of cells treated with **RC-106** (50 μM), BTZ (1 μM) and TG (3 μM) for 12, 12 and 4 h, respectively. Vinculin was used for loading normalization. (**i**) CHOP protein expression in cells untreated and treated with **RC-106** (50 μM), BTZ (1 μM) and TG (3 μM) for 12 h (**RC-106** and BTZ) and 6 h (TG). The images were captured by Nikon Eclipse Ti2 confocal microscope with 60× plan apochromatic oil immersion objective lens. Scale bars are 50 μm and 5 μm for amplified images.

To further our understanding of the relationship between SRs and the terminal UPR pathway, we first compared the activity of our compound with that of two well-known ER stress inducers: thapsigargin (TG) a SERCA inhibitor, and bortezomib (BTZ), a known proteasome inhibitor [46]. The choice of concentrations and exposure time used was based on literature data and on previous results obtained by Real-Time qRT-PCR [45]. To this purpose, cells were treated with the three molecules and the effect on the expression of ER-stress/terminal UPR markers compared. We observed a high upregulation of GRP78 and CHOP in both cell lines following TG exposure, confirming its reliability as an ER stress activator in our model (Figure 1a). We then compared the effect of **RC-106** on GRP78, ATF4 and CHOP mRNA expression with that induced by the reference compounds. Sensors upregulation induced by **RC-106** was fairly similar to that of TG, which was characterized by a strong induction of GRP78 and CHOP expression and a milder induction of ATF4. Conversely, BTZ induced a lower upregulation of the three sensors, especially GRP78.

In Panc-1 cells we observed a higher expression of all sensors after exposure to all compounds compared to untreated controls. Conversely in Capan-1, **RC-106** and TG induced sensors upregulation after only 6 h, whereas BTZ induced a late and lower sensor upregulation (Figure 1b). Remarkably, **RC-106** induced the highest mRNA expression modulation of ATF4 in both cell lines. It also caused the highest cell death in both cell lines, whereas BTZ and TG induced very little or no cell death, respectively (Figure 1c,d). A dose-dependent increase in expression proportional to the increase in **RC-106** concentration was observed in all the sensors evaluated. Independently of the time tested, the highest upregulation of all markers occurred after exposure to **RC-106** 50 μM, concentration matching the IC_50_ observed at the longest exposure time (24 h). (Supplementary Figure S1).

Given that ROS may play an important role in triggering apoptosis and that protein folding is highly redox-dependent, we investigated the ability of **RC-106** to induce ROS production, assayed by hydrogen peroxide (H_2_O_2_) detection. Cell lines were treated for 6, 12 and 24 h with **RC-106** 50 μM, resulting in higher ROS production proportional to the increase in drug exposure time. Of note, the increase in ROS started after 6 h exposure in both cell lines, reaching a maximum value after 24 h (Figure 1e). In parallel, we investigated the effect of **RC-106** on ATF4, which is known to be involved in UPR and antioxidant response [47]. As expected, ATF4 was significantly upregulated after **RC-106** treatment in both cell lines, showing an increase over time superimposable to that of ROS production (Figure 1f). Interestingly, the drug also induced mitochondria depolarization in both cell lines in proportion to apoptosis activation, indicating the triggering of the intrinsic apoptotic pathway (Figure 1g). The highest percentage of apoptotic cells was observed in Panc-1 cell line after 24 h, while Capan-1 cell line showed a significant increase in apoptotic cells after only 6 h exposure (Figure 1d, Supplementary Figure S2). These results are consistent with the increase in ATF4 expression and ROS generation observed, thus highlighting the capability of Panc-1 cell line to survive in conditions of high metabolic stress, as also evidenced by their lower ability to activate programmed cell death with respect to Capan-1. This behavior may be ascribed to multiple reasons, i.e., different expression level of SRs, different growth behaviors and basal stress condition (Supplementary Figure S3), activation/inactivation of other SRs client proteins (IP3R3 receptor, ion channels, Ca^2+^ pumps).

Protein analyses further underlined the ability of our molecule to cause substantial ER stress and to activate terminal UPR signaling, as shown by the increase in GRP78 and CHOP protein expression after **RC-106** treatment. However, ATF4 protein expression was detected also in Panc-1 cells treated with BTZ. Moreover, coherently with its lack of cytotoxicity, TG induced lower CHOP modulation than **RC-106** or BTZ (Figure 1h, Supplementary Figure S4). Finally, we also performed immunofluorescence analysis of CHOP. CHOP expression was induced in both cell lines after all treatments, but with a different localization, i.e., nuclear in Panc-1 cells and more diffuse and extra-nuclear in Capan-1 (Figure 1i).

### 2.2. S1R Post-Transcriptional Silencing Induces ER Stress and Terminal UPR Response in PDAC Cell Lines

**Figure 2:**
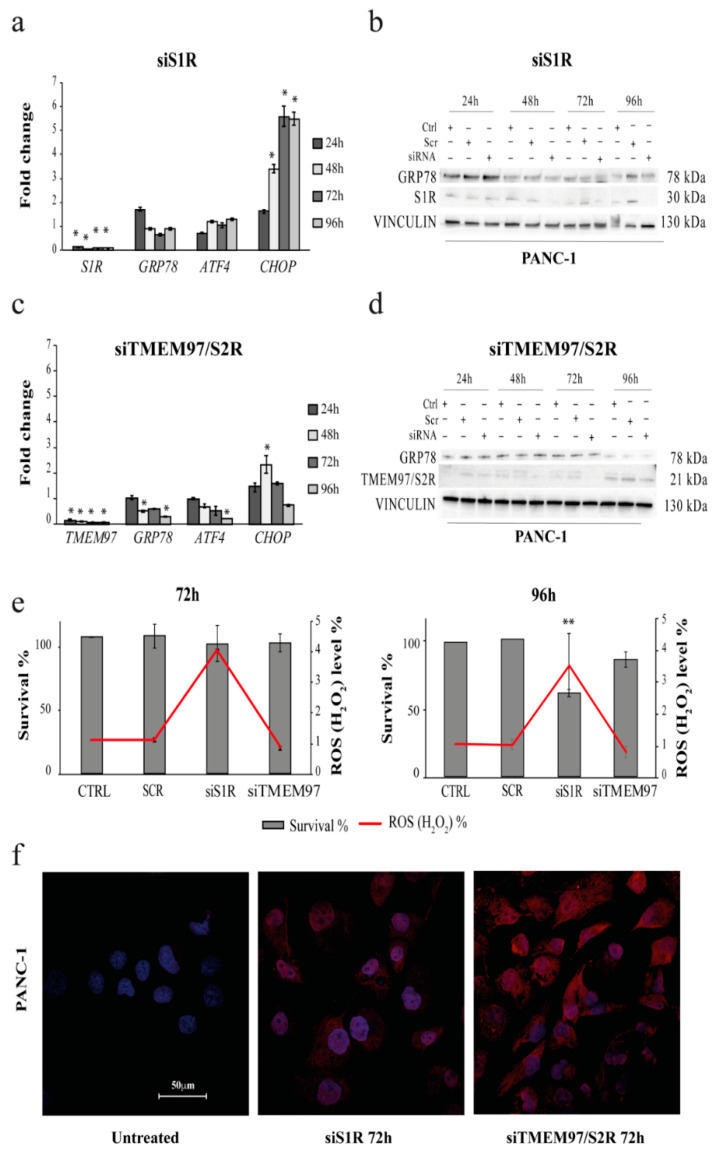
ER sensor modulation after post-transcriptional gene silencing of S1R and TMEM97/S2R in Panc-1 cells. (**a**) Real Time qRT-PCR analysis of mRNA expression levels of S1R, GRP78, ATF4 and CHOP after transient S1R silencing. RNA expression was normalized to GAPDH and HPRT-1. The mRNA expression was relative to the corresponding untreated control at each time-point tested (Fold change = 1). Values are the mean ± SD of two independent experiments. (* *p* ≤ 0.05 vs. CTRL). (**b**) Western blot of S1R and GRP78 in Panc-1 cells silenced with S1R siRNA. Vinculin was used as loading control. (**c**) Real Time qRT-PCR analysis of mRNA expression levels of TMEM97/S2R, GRP78, ATF4 and CHOP after transient TMEM97/S2R silencing. RNA expression was normalized to GAPDH and HPRT-1. The mRNA expression was relative to the corresponding untreated control at each time point tested (Fold change = 1). Values are the mean ± SD of two independent experiments. (* *p* ≤ 0.05 vs. CTRL). (**d**) Western blot of TMEM97/S2R and GRP78 in Panc-1 cells silenced with TMEM97/S2R siRNA. Vinculin was used as loading control. (**e**) ROS-Glo^TM^ H_2_O_2_ assay and CellTiter-Glo^Ⓡ^ Luminescent Cell Viability Assay multiplex. Cells were seeded in a 96-well plate and silenced for 72 h and 96 h with S1R and TMEM97/S2R siRNA, respectively. H_2_O_2_ substrate was added 6 h before the time of dosing. After incubation, an aliquot of media was removed from the assay well for ROS detection. CellTiter-Glo^®^ was added to the original assay wells for cell survival evaluation. Values are the mean ± SD of two independent experiments. (** *p* ≤ 0.01 vs. CTRL). (**f**) Confocal microscopy immunofluorescence of CHOP (red) expression in cells untreated or silenced with S1R and TMEM97/S2R siRNAs. Nuclei are stained with DAPI (blue). Cell images were captured by Nikon Eclipse Ti2 confocal microscope with 60× plan apochromatic oil immersion objective lens. Scale bars are 50 μm.

Then, to confirm the direct correlation between the effect exerted by **RC-106** on ER stress triggering and on terminal UPR response, we performed post-transcriptional silencing of both SRs in both PDAC cell lines. In Panc-1 cell line, S1R and TMEM97/S2R were completely silenced (Supplementary Figures S5–S7). Notably, while silencing S1R, we noticed that GRP78 (mRNA and protein expression) and ATF4 mRNA expression levels did not change significantly (Figure 2a,b). Simultaneously, S1R silencing led to a significant upregulation of CHOP mRNA expression, a finding also confirmed by immunofluorescence where nuclear CHOP localization was clearly visible (Figure 2f). Consistent with this observation, significant ROS expression was induced in S1R-silenced cells together with a decrease in cell survival after 96 h of transient gene silencing (Figure 2e). Conversely, TMEM97/S2R silencing induced a gradual downregulation of GRP78 (mRNA and protein expression) and significant CHOP expression only after 48 h exposure to the siRNA (Figure 2c,d). Moreover, we observed high CHOP protein upregulation, albeit mainly cytoplasmatic, after TMEM97/S2R silencing (Figure 2f). Notably, TMEM97/S2R silencing induced a significant downregulation of ATF4, which probably led to a lower upregulation of CHOP (Figure 2c). Interestingly, TMEM97/S2R silenced cells did not show ROS expression or a high percentage of cell death (Figure 2e).

**Figure 3:**
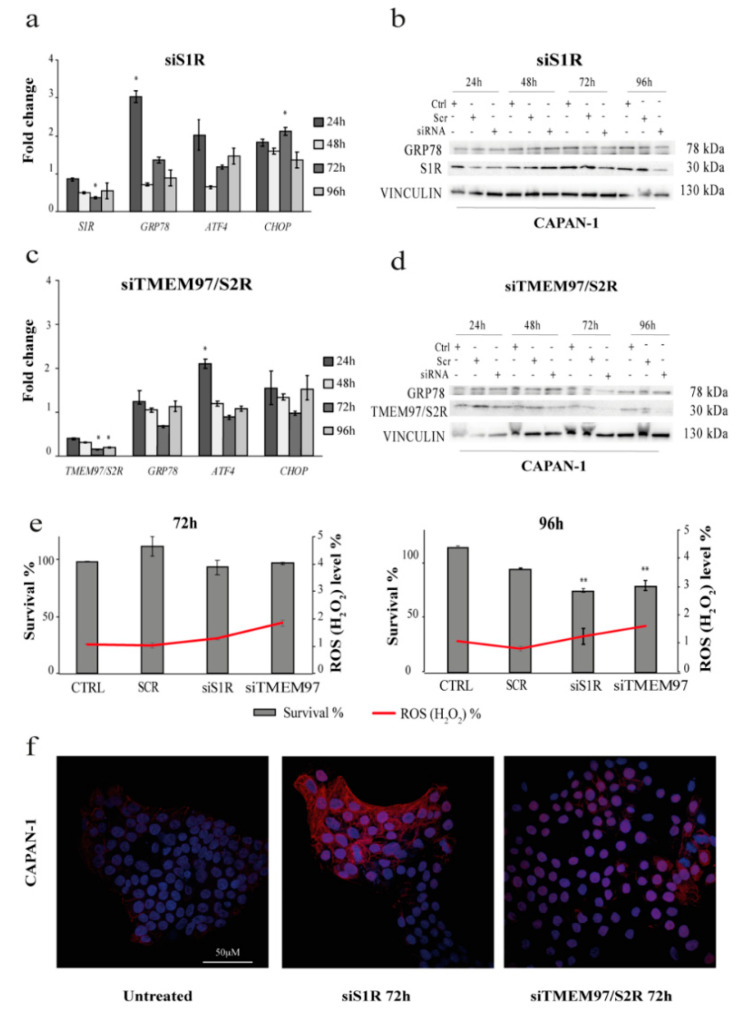
ER sensor modulation after post-transcriptional gene silencing of S1R and TMEM97/S2R in Capan-1 cells. (**a**) Real Time qRT-PCR analysis of mRNA expression levels of S1R, GRP78, ATF4 and CHOP after transient S1R silencing. mRNA expression was normalized to GAPDH and HPRT-1. The mRNA expression was relative to the corresponding untreated control at each time-point tested (Fold change = 1). Values are the mean ± SD of two independent experiments (* *p* ≤ 0.05 vs. CTRL). (**b**) Western blot of S1R and GRP78 expression in Panc-1 cells silenced with S1R siRNA. Vinculin was used as loading control. (**c**) Real-Time qRT-PCR analysis of mRNA expression levels of TMEM97/S2R, GRP78, ATF4 and CHOP after transient TMEM97/S2R silencing. mRNA expression was normalized to GAPDH and HPRT-1. The mRNA expression was relative to the corresponding untreated control at each time-point tested (Fold change = 1). Values are the mean ± SD of two independent experiments (* *p* ≤ 0.05 vs. CTRL). (**d**) Western blot of TMEM97/S2R and GRP78 expression in Panc-1 cells silenced with TMEM97/S2R siRNA. Vinculin was used as loading control. (**e**) ROS-Glo^TM^ H_2_O_2_ assay and CellTiter-Glo^®^ Luminescent Cell Viability Assay multiplex. Cells were seeded in 96-well plate and silenced for 72 h and 96 h with S1R and TMEM97/S2R siRNA, respectively, H_2_O_2_ substrate was added 6 h before the time of dosing. After incubation, an aliquot of media was removed from the assay well for ROS detection. CellTiter-Glo^®^ was added to the original assay wells for cell survival evaluation. Values are the mean ± SD of two independent experiments. ** *p* ≤ 0.01 vs. CTRL. (**f**) Confocal microscopy immunofluorescence of CHOP (red) expression in cells untreated or silenced with S1R and TMEM97/S2R siRNAs. Nuclei are stained with DAPI (blue). Cell images were captured by Nikon Eclipse Ti2 confocal microscope with 60× plan apochromatic oil immersion objective lens. Scale bars are 50 μm.

Although we did not obtain the complete switch-off of S1R and TMEM97/S2R (about 80% decreased expression) in Capan-1 cell line, the partial shutdown of both receptors resulted in a modulation of ER stress sensor markers (Supplementary Figures S6 and S7). The S1R downregulation effect on CHOP, GRP78 (mRNA and protein) and ATF4 was similar to that observed in Panc-1 cell line, thus confirming our previous observations (Figure 3a,b). Immunofluorescence showed that the S1R downregulation led to CHOP upregulation in Capan-1 cells, albeit with less evident nuclear protein staining than in Panc-1 (Figure 3f). Consistent with the milder CHOP upregulation, we did not observe significant ROS expression in S1R-silenced Capan-1 cells, which resulted in a poorer cell survival than Panc-1 cell line (Figure 3e). Conversely, TMEM97/S2R silencing led to an expression modulation similar to that seen in Panc-1 cells, but data were not significant for any of the sensors studied (Figure 3c,d). An upregulation of CHOP expression observed at protein level was milder than that caused by S1R silencing (Figure 3f).

### 2.3. **RC-106** Effect Relies on the Modulation of Both SRs

**Figure 4:**
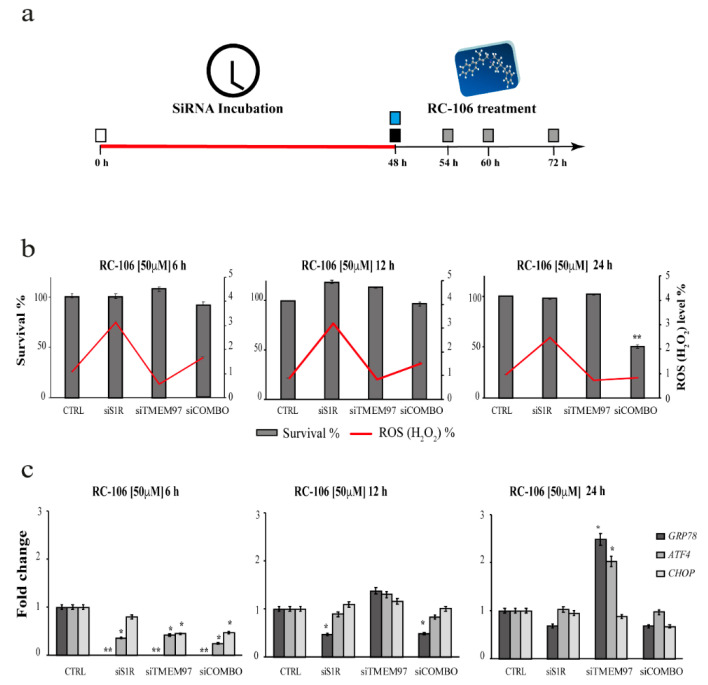
**RC-106** effect on SRs-silenced PANC-1 cells (**a**) Scheme of experiment: plating of cells and start of transfection at 0 h. Transfection complexes remained in the growth medium for 48 h. After 48 h, the medium was changed for one without complexes and cells were treated with **RC-106** 50 μM for 6,12 and 24 h. Cell viability, ROS generation and ER stress sensors up-regulation analyses were performed at 54, 60 and 72 h. Symbols: red line: transfection; white rectangle: plating cells and adding transfection complexes; black rectangle: medium exchange; blue rectangle treating silenced cell with **RC-106**; red rectangle: ROS assay, CellTiter-Glo^®^ assay and real time RT qPCR. (**b**) ROS-Glo^TM^ H_2_O_2_ assay and CellTiter-Glo^®^ Luminescent Cell Viability Assay multiplex. Cells were seeded in 96-well plate and silenced for 48 h and 72 h with S1R and TMEM97/S2R siRNA, respectively, H_2_O_2_ substrate was added 6 h before the time of dosing. After incubation, an aliquot of media was removed from the assay well for ROS detection. CellTiter-Glo^®^ was added to the original assay wells for cell survival evaluation. Control is represented by cells transfected with scrambled siRNA and treated with **RC-106** 50 μM at the corresponding time point. Values are the mean ± SD of two independent experiments. ** *p* ≤ 0.01 vs. control. (**c**) Real Time qRT-PCR analysis of mRNA expression levels of GRP78, ATF4 and CHOP after transient S1R and TMEM97/S2R silencing both alone and together. mRNA expression was normalized to GAPDH and HPRT-1. The mRNA expression was relative to the control, represented by cells transfected with scrambled siRNA and treated with **RC-106** 50 μM at the corresponding time point. (Fold change = 1). Values are the mean ± SD of two independent experiments. (* *p* ≤ 0.05, ** *p* ≤ 0.01 vs. CTRL).

To investigate if **RC-106** effect might be also driven by mechanisms independent from SRs, we conducted functional studies on PANC-1 cell line. We performed such studies only on these cells due to the inability to completely silence SRs in Capan-1 cell line. In particular, we transiently silenced SRs, both separately and simultaneously, and we assayed cell viability, ROS generation and ER stress sensors up-regulation. Transient silencing was performed adding the transfection complex at the time of plating the cells and leaving it in the growth medium for 48 h. Then, medium was changed, and cells were treated with **RC-106** 50 μM for 6, 12 and 24 h (Figure 4a). Silencing S1R alone and treating cells with **RC-106** we observed, as expected, an increase in ROS generation at all the exposure time tested. Moreover, also ER-stress markers were modulated, showing at the first time points (6–12 h) a significantly lower expression as compared to the control and increasing over-time. Additionally, no decrease in cell viability was reported (Figure 4b,c). Silencing TMEM97/S2R alone and treating cells with **RC-106** we observed no increase in ROS generation at all the exposure time tested. Furthermore, ER-stress sensors’ expression increased over time as a consequence of **RC-106** activity towards S1R. In particular, after 6 h of exposure to **RC-106**, sensors’ expression levels were significantly lower as compared to the control, reaching their maximum after 24 h of exposure to the compound. Furthermore, no decrease in cell viability was reported (Figure 4b,c). Finally, silencing simultaneously both SRs and treating cells with **RC-106** we observed a slight increase in ROS generation after 6 h exposure, that progressively decreased. Likewise S1R silenced cells, ER-stress markers expression increased over time, being at the first time points (6–12 h), significantly less expressed as compared to the control. Regarding cell viability, a significant reduction in cell viability was observed only after 24 h exposure to **RC-106**.

### 2.4. Cytotoxicity of **RC-106**-Nab-Paclitaxel Drug Combination

**Figure 5:**
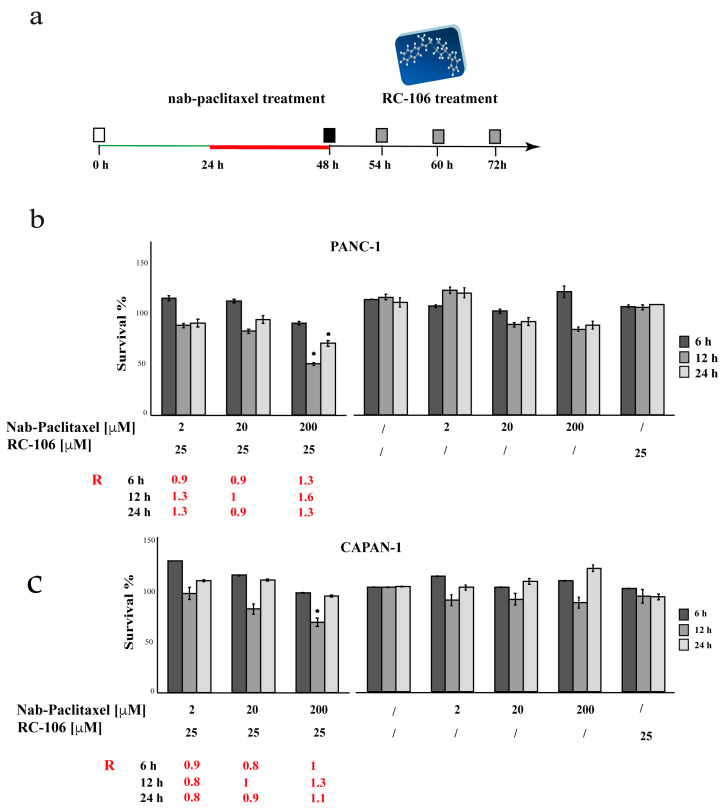
Drug combination effect (**a**) Scheme of experiment: plating of cells at 0 h. 24 h after cell plating, nab-PTX treatment for 24 h. Medium was changed prior to expose cells to **RC-106**. **RC-106** treatment for 6, 12 and 24 h. Cell viability analyses were performed at 54, 60 and 72  h. Symbols: green line: cell seeding without drugs; red line: nab-PTX treatment; black line: **RC-106** treatment; white rectangle: cell plating; black rectangle: medium wash-out; grey rectangles: experiments read-out. (**b**) Cell survival of Panc-1 and Capan-1 cell lines after treatment with the three most promising drug combinations and after the treatment with the two drugs alone. Cells were exposed to drug combinations and to drugs alone for 6, 12, and 24 h. MTS assay was used to determine cell viability. Values are the mean ± SD of two independent experiments. The “synergistic ratio” (**R**) values for each drug combination, with *R* < 1 indicating antagonism, 0 and *R* > 1 indicating synergism. * *p* < 0.05 represent significant increased activity of the combination compared to single drug treatments. (**b**,**c**) are reported the most effective drug combinations.

The interaction between **RC-106** and nab-paclitaxel (nab-PTX) activity was investigated in PC cell lines using the following scheme: cells were exposed to nab-paclitaxel for 24 h (corresponding to the drug half-life), then, cells were exposed to **RC-106** for 6, 12 and 24 h (nab-PTX → **RC-106**). The first drug was washed out before adding the second (Figure 5a). The drugs were assumed to act as independent variables. To evaluate the interaction between drugs, the synergistic ratio (R), was calculated. In Figure 5b,c are reported the most effective drug combinations. In detail, the sequences **RC-106** 25 μM preceded by nab-PTX 2, 20 and 200 μM represented the most effective combinations, independently from the exposure time, causing an additive effect in both Panc-1 (R ranging from 0.9 to 1.6) and Capan-1 (R ranging from 0.8 to 1.1).

## 3. Discussion

The identification of novel druggable targets could open up new frontiers for the discovery and development of effective therapies for cancers with poor clinical outcome, such as PDAC. With this concept in mind, we focused on ER stress and terminal UPR activation, known to be involved in cancer pathogenesis. In particular, the UPR emerged as a key pathway in both tumor-promotion and tumor-suppression [48]. In a previous paper, we hypothesized a role of ER stress gatekeepers for SRs in light of their localization, chaperone function and interactions with a great number of proteins. In the present work, we demonstrated that SRs modulation results in ER stress induction, probably as a result of unfolded protein accumulation which, in turn, leads to increased ROS generation followed by cell death due to apoptosis activation.

We first investigated the triggering of ER stress in two PDAC cell lines after exposure to **RC-106**, a pan SR modulator identified by our team [42] and thought to have an effect on ER stress induction. In particular, we focused on the effect of **RC-106** on GRP78, ATF4 and CHOP, the key players in terminal UPR triggering. Whilst GRP78 represents one of the main ER stress sensors, ATF4 and CHOP play a pivotal role in terminal UPR transduction [49,50]. The impact of **RC-106**, the proteasome inhibitor BTZ and the SERCA pump inhibitor TG, commonly used in in vitro ER stress studies, were evaluated in PC cells and their effects compared. As expected, both TG and BTZ induced initial GRP78 upregulation followed by the induction of terminal UPR, as confirmed by CHOP upregulation at both transcriptional and protein level. Similar observations were made for **RC-106**, which induced an early upregulation of GRP78 comparable to that of TG, thus demonstrating its ability to induce ER stress. **RC-106** also induced greater CHOP overexpression than the two reference compounds, thus confirming its ability to affect the adaptive UPR response by promoting the switch of this adaptive pathway to a cell death message. Notably, this mechanism was probably enhanced by the proteasome inhibition activity we previously reported for **RC-106** [51]. This finding was further confirmed by the higher cell death induced by **RC-106** compared to the reference compounds.

Literature data suggest that, as S1R regulates Ca^2+^ influx from MAMs to mitochondria, promoting mitochondrial metabolism and ROS generation, alterations of the mitochondrial Ca^2+^ signaling caused by a functional inhibition of S1R may induce ROS production [18,32,52]. Moreover, it has been shown that TMEM97/S2R is involved in the regulation of lipid transport/metabolism and that lipid and glucose metabolism are finely regulated at MAMs. On the basis of these findings, we oriented our research to study the effect of our pan-SRs modulator on ER stress, proteasome activity and redox state of the cell through the induction of a ROS wave.

The data we obtained revealed a strong production of ROS when PC cell lines were exposed to **RC-106**. This was probably due to the simultaneous action of **RC-106** on both S1R and TMEM97/S2R, which impaired the redox homeostasis of the ER, fundamental for the survival of cells. Both cell lines reacted to this condition with a significant induction of ATF4 expression, a transcription factor regulating several UPR target genes, including the pro-apoptotic transcription factor CHOP and others involved in the antioxidant response [53]. In particular, we observed a substantial ROS production and higher ATF4 upregulation in Panc-1 cells, which appeared to be more resistant to **RC-106**.

To further corroborate the potential of SRs as druggable targets for ER stress-activated terminal UPR induction, we performed transient post-transcriptional silencing of S1R and TMEM97/S2R. Despite difficulties encountered in the gene silencing of these markers caused by the lack of more specific commercially available products, we identified a direct correlation between SRs and the UPR transducers ATF4, GRP78 and CHOP. In particular, both **RC-106** treatment and transient S1R gene silencing caused a significant upregulation of CHOP, together with an early upregulation of GRP78 followed by its downregulation, which is consistent with its anti-apoptotic role. The high CHOP upregulation was consistent with significant ROS generation and increased cell death detected after 96 h of transient S1R silencing. TMEM97/S2R gene silencing caused a downregulation of GRP78 and ATF4, which, in turn, prevented notable CHOP upregulation. Interestingly, TMEM97/S2R silenced cells did not show ROS generation or high cell death. These findings warrant further investigation of the role of TMEM97/S2R in UPR modulation, especially in terms of its potential relationship with stress sensing and redox regulation.

In addition, to investigate whether the effect of our molecule could be ascribed also to SRs-independent mechanisms, we performed functional studies treating Panc-1-SRs-silenced cells with **RC-106**. Cell viability, ROS generation and ER-stress sensors modulation were measured in **RC-106** treated cells, where SRs were silenced both alone and simultaneously.

These experiments confirmed that ROS generation increase is attributable to S1R modulation, observed both by treating cells with **RC-106** and by transient post-transcriptional gene silencing. TMEM97/S2R, on the other hand, seems not to be involved in redox mechanisms, in fact the treatment with **RC-106** of cells expressing this receptor alone did not caused any increase in ROS production. These results also confirm the data obtained in the functional studies previously described. The same observations seem to be true for ER-stress sensors’ modulation. Functional experiments revealed indeed the direct involvement of S1R receptor in ER-stress sensing, but only a marginal role of TMEM97/S2R. The lack of significant GRP78 and ATF4 up-regulation in S1R-silenced-Panc-1 cells may be due to the triggering of a different branch of the UPR machinery. In fact, ATF4 and CHOP are activated by the signaling cascade known to regulate cell transcriptional activity, driven by PERK, one of the three main UPR sensors. Being TMEM97/S2R involved in lipid homeostasis and cholesterol biosynthesis, it probably triggers the terminal UPR activating the UPR branch that regulates lipid synthesis and protein secretion, driven by IRE-1α [54]. Relative to S1R we observed that, S1R-silenced cells treated with **RC-106,** showed moderate increase of ER stress sensors expression over time, probably due to the lack of the receptor it-self. Moreover, in TMEM97/S2R-silenced-cells treated with **RC-106,** we observed a substantial increase in ER stress sensors modulation, attributable to the modulatory activity of **RC-106** on S1R.

Relative to cell survival, since we referred to cells exposed to the scrambled siRNA and treated with **RC-106** as control, we did not observed significant variations in Panc-1 cells silenced with S1R or TMEM97/S2R siRNA alone. However, when both SRs were lacking, we observed, at the highest exposure time, a decrease in cell survival. In light of our previous results relative to **RC-106** activity characterization, we can hypothesize that the increase in **RC-106** efficacy observed could be due to its effect on the proteasome activity.

Overall, the results from the present study provide an in-depth overview of the involvement of SRs in ER stress-induced apoptosis known as terminal UPR, mediated by ROS generation, which could be exploited for the development of new therapeutic options. Our data confirm that SRs serve as ER stress gatekeepers by modulating cell behavior in response to ER stress, thus suggesting the potential therapeutic application of pan-SRs to trigger terminal UPR to induce cancer cell death.

**Figure 6:**
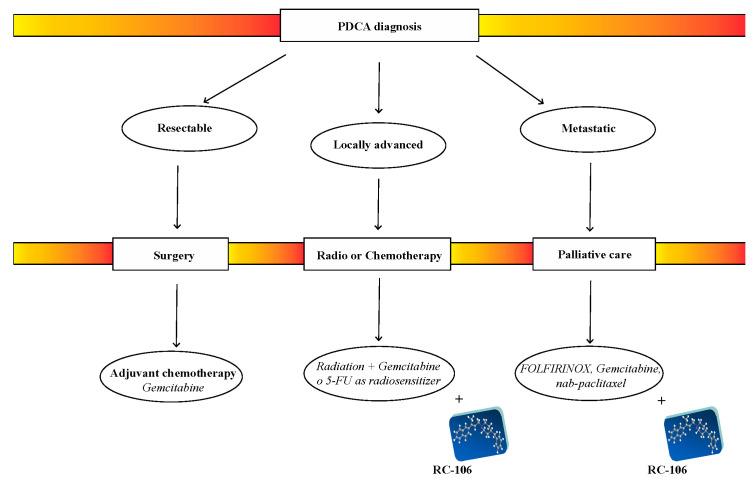
Theoretical treatment algorithm for pancreatic cancer.

Finally, since most PDAC patients receive treatments such as chemotherapy and/or radiotherapy having a minimal impact on survival due to the intrinsic resistance to apoptosis of this tumor [7], we evaluated the in vitro effect of **RC-106** treatment in combination with nab-paclitaxel (Abraxane). This drug is used in clinical practice for the treatment of locally advanced and metastatic tumors in combination with Gemcitabine, however this treatment option is highly dependent on patient’s performance status [55] (Figure 6). Our preliminary investigation focused on the drug sequence nab-PTX → **RC-106** and different combinations of drug concentrations and exposure times were assayed. The sequences **RC-106** 25 μM preceded by nab-PTX 2, 20 and 200 μM represented the most effective combinations, independently from the exposure time, resulting in an additive effect in both Panc-1 and Capan-1 cell lines. Despite the lack of a synergistic effect, we believe that drug combinations studies using **RC-106** deserve to be deepened. UPR triggering is indeed known to be involved in drug resistance mechanisms, thus potentially, targeting this pathway through the use of a pan-SRs modulator may represent a strategy to overcame resistance to chemotherapy.

Based on the positive results obtained, **RC-106** could be proposed as a model compound to obtain proof of concept of the in vivo efficacy of SR modulators in cancer treatment, including pancreatic cancer. Looking forward, this work could be expanded to find an effective drug candidate for PC.

Finally, in light of recent publications relating to SARS-CoV-2, we would like to draw attention to the potential usefulness of SR modulators as antiviral agents thanks to their ability to interfere with the protein synthesis machinery driven by terminal UPR activation and proteasome inhibition [21,56,57].

## 4. Materials and Methods

### 4.1. Cell Lines and Chemicals

**Scheme 1:**
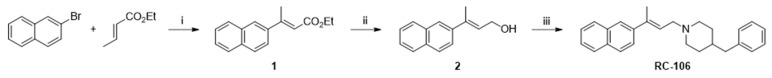
Synthesis of **RC-106**. Reagents and reaction conditions: (i) Pd EnCat (0.01 equiv.), TEAC (2 equiv.), AcONa (2 equiv.), dry DMF, 105 °C, yield 58%; (ii) LiAlH4 (1M in THF, 1 equiv.), dry Et2O, 0 °C, yield 82%; (iii) PPh3 (1.5 equiv.), NBS (1.4 equiv.), 4-benzylpiperidine (1.2 equiv.), Et3N (2 equiv.), THF, −18 °C, yield 65%.

PDAC cell lines Panc-1 and Capan-1 were purchased from ATCC (ATCC, Manassas, VA, USA). Both cell lines were cultured in DMEM/Ham’s F12 (1:1; EuroClone S.p.a, Milan, Italy) supplemented with FBS (10%; EuroClone S.p.a, Milan, Italy); glutamine (2 mM; EuroClone S.p.a, Milan, Italy) and insulin (10 µg/mL; Sigma Aldrich, St. Louis, MO, USA). Cells were incubated in a humidified atmosphere containing 5% CO_2_ at 37 °C and routinely tested for mycoplasma. **RC-106** was synthesized by us applying an easy to handle synthetic route, previously described by our group [51] and outlined in Scheme 1. Briefly, **RC-106** was prepared starting from 2-bromonaphthalene via Heck reaction, subsequent reduction of the ester moiety to alcohol and a one-pot activation of the hydroxyl group and nucleophilic substitution with 4-benzylpiperidine. **RC-106** was obtained in sufficient purity (>98%) and amount (hundred mgs-scale) for the biological investigation. Purity of the final product was assessed by UPLC-UV-ESI/MS analysis, employing the same method reported in our previous publication [51].

### 4.2. Drug Combinations

In the sequential treatment scheme, cell exposed for 24 h to nab-paclitaxel 2, 20 and 200 μM where then treated with **RC-106** 12.5, 25 and 50 μM for 6, 12 and 24 h. The first drug was washed out before adding the second and the cytotoxic effect was evaluated immediately after the end of drugs exposure. **RC-106** drug concentrations were chosen basing on previous results, while nab-PTX concentrations where determined using as a starting point the plasma peak concentration (20 μM).

### 4.3. Drug Interaction Analysis

Several methods have been proposed to evaluate the interaction between drugs [58], the most appropriate in case of low cytotoxic effect or no dose-response curves, as for nab-paclitaxel, is the Kern’s method [59], subsequently modified by Romanelli. Briefly, the expected cell survival (S*exp*), defined as the product of the survival observed with drug A alone and the survival observed with drug B alone) and the observed cell survival (S*obs*) for the combination of A and B were used to build-up the synergistic ratio (R): R = S*exp*/S*obs*. An R ≤ 1 (additive effect) indicates the absence of synergism or antagonism. Synergism was defined as any value of R greater than unity.

### 4.4. MTS Assay

Cytotoxicity assays were performed using CellTiter 96^®^ AQueous One Solution Cell Proliferation Assay (Promega, Milan, Italy). Cells seeded onto a 96-well plate were exposed to increasing concentrations of the drugs. The effect of the treatments was evaluated after a 6, 12 and 24 h exposure. Two independent experiments were performed in octuplicate. The optical density (OD) of treated and untreated cells was determined at a wavelength of 490 nm using a fluorescence plate reader. Dose response curves were created using Excel software and IC_50_ values were determined graphically from the plot.

### 4.5. CellTiter-Glo^®^ Luminescent Cell Viability Assay

Cell viability of silenced and naïve cells was measured using a luminescent cell viability assay (Promega, Milan, Italy). Briefly, cells were plated in a 96-well opaque culture plate (BD Falcon, Corning, Somerville, MA, USA), CellTiter-Glo^®^ reagent was added to each well and the luminescence signal was read after 30 min with the GloMax^®^ 96 microplate luminometer (Promega, Milan, Italy).

### 4.6. ROS Assay

The ROS-Glo^TM^ H_2_O_2_ assay (Promega, Milan, Italy) was used to detect hydrogen peroxide (H_2_O_2_) levels in cell culture according to the manufacturer’s instructions. The ROS assay was performed by plating 1 × 10^4^ cells into each well of a 96-well plate. Cells were treated with compounds, incubated with H_2_O_2_ substrate solution for 6 h, after which ROS-Glo detection solution was added. Luminescence units were measured using GloMax^®^ 96 microplate luminometer (Promega, Milan, Italy). Values were normalized to protein concentration.

### 4.7. Annexin V assay

Treated and untreated cells were washed in PBS 1X and then incubated with 25 μL/mL Annexin V-FITC in binding buffer (Thermo Fisher Scientific, Waltham, MA, USA) for 15 min at 37 °C in a humidified atmosphere in the dark. Immediately before flow cytometric analysis, 5 μg/mL of propidium iodide was added to discriminate between apoptotic and necrotic cells.

### 4.8. TUNEL Assay

Cells were fixed in 1% formaldehyde in PBS 1X on ice for 15 min, suspended in 70% ice-cold ethanol and stored overnight at −20 °C. Cells were then washed twice in PBS and permeabilized with a solution of 0.1% Triton X-100 in PBS for 5 min at room temperature. Thereafter, samples were incubated in a 50 µL solution containing TdT and FITC conjugated dUTP deoxynucleotides 1:1 (Roche Diagnostic GmbH, Mannheim, Germany) in a humidified atmosphere for 90 min at 37 °C in the dark, washed in PBS, counterstained with propidium iodide (2.5 μg/mL, MP Biomedicals, Verona, Italy) and RNAse (10 kU/mL, Sigma-Aldrich, St. Louis, MO, USA) for 30 min at 48 °C in the dark and analyzed by flow cytometry.

### 4.9. JC-1 Assay

Mitochondrial membrane potential was assessed using MitoPT^TM^ JC-1 Assay Kit (ImmunoChemistry Technologies, Bloomington, MN, USA), according to the manufacturer’s protocol for flow cytometry analysis. Briefly, cells were seeded in T25 flasks (5 × 10^5^ cells/well) and cultured overnight. After treatment with the drugs, cells were collected and centrifuged at 1200 rpm ×  5 min. Cell pellets were resuspended in JC-1 working solution and incubated at 37 °C for 20 min in the dark. CCCP was used as positive control. Samples were subsequently analyzed detecting JC-1 aggregates with red fluorescence (590 nm emission) or the monomeric form with green fluorescence (527 nm emission).

### 4.10. RealTime RT-qPCR

TRIzol reagent (Life Technologies, Carlsbad, CA, USA) was used, in accordance with the manufacturer’s instructions, for total cellular RNA extraction. RNA was quantified by the Nanodrop MD-1000 spectrophotometer system. iScript cDNA Synthesis kit (Bio-Rad Laboratories, Hercules, CA, USA) was used to perform reverse transcription reactions, in particular 400 ng of total RNA were retrotranscribed in 20 μL of nuclease-free water. Real-Time PCR was performed by 7500 Fast Real-Time PCR system (Applied Biosystems, Foster City, CA, USA) and the expression of SIGMAR1, TMEM97, GRP78, ATF4, and CHOP genes were detected by TaqMan assays. Reactions containing 40 ng of cDNA template, TaqMan universal PCR Master Mix (2X), and selected TaqMan assays (20X) were carried out in triplicate at a final volume of 20 μL. Samples were maintained at 50 °C for 2 min, then at 95 °C for 10 min followed by 40 amplification cycles at 95 °C for 15 s, and at 60 °C for 30 s. Gene expression was normalized to the endogenous genes GAPDH and HPRT-1.

### 4.11. Western Blot

Cell proteins were extracted with RIPA lysis and extraction buffer (Thermo Fisher Scientific, Waltham, MA, USA). Mini-PROTEAN TGXTM precast gels (4–20%; BIO-RAD, Hercules, CA, USA) were run using Mini-PROTEAN Tetra electrophoresis cells and then electroblotted by Trans-Blot Turbo^TM^ Mini PVDF Transfer Packs (BIO-RAD, Hercules, CA, USA). Membranes were blocked with 5% non-fat dry milk in TBST 1X for 1 h at room temperature and incubated with primary antibodies overnight at 4 °C. Membranes were then incubated with proper HRP-conjugated secondary antibodies. The antibody-antigen complexes were detected with Clarity Max Western ECL Substrate (BIO-RAD, Hercules, CA, USA). The following primary antibodies were used, diluted as indicated: anti-sigma receptor (S18), 1:500 (sc-22948; Santa Cruz Biotechnology Inc., Dallas, TX, USA), anti-TMEM97, 1:500 (#PA5-23003; Thermo Fisher Scientific, Waltham, MA, USA), anti-Bip, 1:1000 (#3177; Cell Signaling, Danvers, MA, USA), anti-Atf4, 1:1000 (#11815; Cell Signaling, Danvers, MA, USA) and anti-Chop, 1:1000 (#2895; Cell Signaling, Danvers, MA, USA) anti-pAkt, 1:1000 (#9271, Cell Signaling, Danvers, MA, USA). Anti-vinculin, 1:1000 (sc-5573; Santa Cruz Biotechnology Inc., Dallas, TX, USA) was used as a housekeeping. Quantity One Software was used for densitometry analysis.

### 4.12. Immunofluorescence

Cells were fixed in PFA 4% for 20 min at room temperature, blocked with 5% FBS plus 0.1% Triton X-100 in PBS 1X and incubated overnight at 4 °C with primary antibody (anti-CHOP (L63F7), #2895; Cell Signaling, Danvers, MA, USA) diluted 1:200. After washing with PBS 1X 3 times, slides were incubated for 1 h at room temperature with secondary goat anti-mouse Alexa Fluor 546 (1:250; Life Technologies, Carlsbad, CA, USA). Images were taken with a Nikon A1 confocal laser scanning microscope, equipped with a 60×, 1.4 NA lens and with 405 and 561 nm laser lines.

### 4.13. Post-Transcriptional Gene Silencing

Post-transcriptional gene silencing was performed by the reverse transfection method. Cells were seeded at a density of 1.75 × 10^4^ in a T25-cm^2^ flask on the day of transfection. To allow complex formation, 700 µL of Opti-MEM GlutaMax medium (Invitrogen, Carlsbad, CA, USA) without antibiotics were placed in a sterile tube with 10 µM siRNA stock solution (Life Technologies, Carlsbad, CA, USA) and TransIT-X2 (Mirus Bio LLC, Madison, WI, USA) in a 1:1 ratio. Tubes were incubated for 20 min on ice and finally transferred into the flasks. Silencer select^®^ siRNAs (SIGMAR1, s20086 and TMEM97, s26204, Life Technologies, Carlsbad, CA, USA) and validated Negative Universal ControlTM (Invitrogen, Carlsbad, CA, USA) final concentrations ranged from 40 nM to 80 nM. Cells were incubated for 24 to 96 h with the complexes and assayed for knockdown of target gene expression.

### 4.14. Statistical Analysis

All experiments were performed at least in duplicate. Statistical analysis was carried out using GRAPH PAD PRISM 5.0 software by applying the Student t test for 2-group comparisons. Differences were considered significant at *p* < 0.05 and *p* < 0.01.

## 5. Conclusions

**Figure 7:**
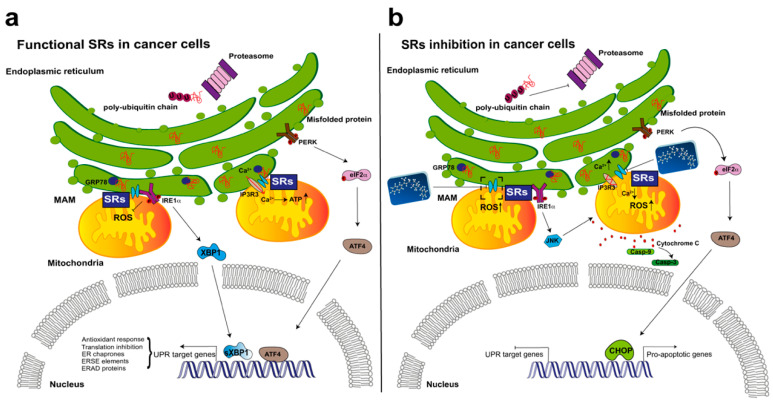
Model depicting how Sigma receptor inhibition may induce cell death in cancer cells. (**a**) Cancer cells have to deal with a large load of unfolded proteins due to unfavorable microenvironmental conditions. Unfolded proteins are removed by proteasome degradation. Accumulation of unfolded proteins in the ER also induces main UPR sensor activation (PERK, IRE1a, ATF6). IRE1 splices XBP1 to an active transcription factor that mediates adaptation of the ER to high secretory demand. PERK phoshorylates eIF2a which, in turn, activates ATF4, a transcription factor involved in the antioxidant response. SRs contribute to cell survival thanks to their chaperone function, stabilizing IRE1a and regulating Ca^2+^ homeostasis through the stabilization of the IP3R3 receptor. (**b**) SR modulators inhibit IRE1a stabilization, causing high ROS generation, and impair Ca^2+^ homeostasis, leading to cytochrome C release and apoptosis induction. The inhibition of the pro-survival function of these receptors induces chronic ER stress, causing UPR to shift from an adaptation pathway to a cell death signal (terminal UPR). The activation of the terminal UPR induces apoptosis mediated by the activation of the transcription factor CHOP, leading to cancer cell death.

Since ER stress was first discovered, stress sensors and stress response mechanisms have been intensively studied, and the importance of ER stress response in human health and disease is now widely acknowledged. In particular, the tumor suppressive function of the UPR pathway is triggered in conditions of chronic or unsustainable ER stress where UPR sensors, not capable of properly counteracting the imbalance of protein homeostasis, induce cell death. It thus becomes intuitive that, chaperones residing at the interface between ER and mitochondria (MAMs), such as SRs, must play an essential role in determining the final outcome of the UPR response, making them appealing targets for anticancer therapy. Our results underlined the central role of SRs in activating ER-stress induced apoptosis. In particular, ROS generation and the upregulation of the main terminal UPR transcription factor, CHOP, highlighted the key role of SRs in activating the terminal UPR pathway, thus identifying pan-SR ligands as candidates for targeting the UPR in pancreatic cancer (Figure 7a,b).

## Supplementary Materials

Supplementary Materials can be found at https://www.mdpi.com/1422-0067/21/23/9012/s1.

## Declarations

Authors declare that the data discussed in the present manuscript are part of a PhD thesis by the author M.C.
