# Supplementary figures and images for: Pan-Sigma Receptor Modulator RC-106 Induces Terminal Unfolded Protein Response In In Vitro Pancreatic Cancer Model
Source: Int J Mol Sci. 2020 Nov 27;21(23):9012. doi: 10.3390/ijms21239012 (PMC7734580; doi:10.3390/ijms21239012)

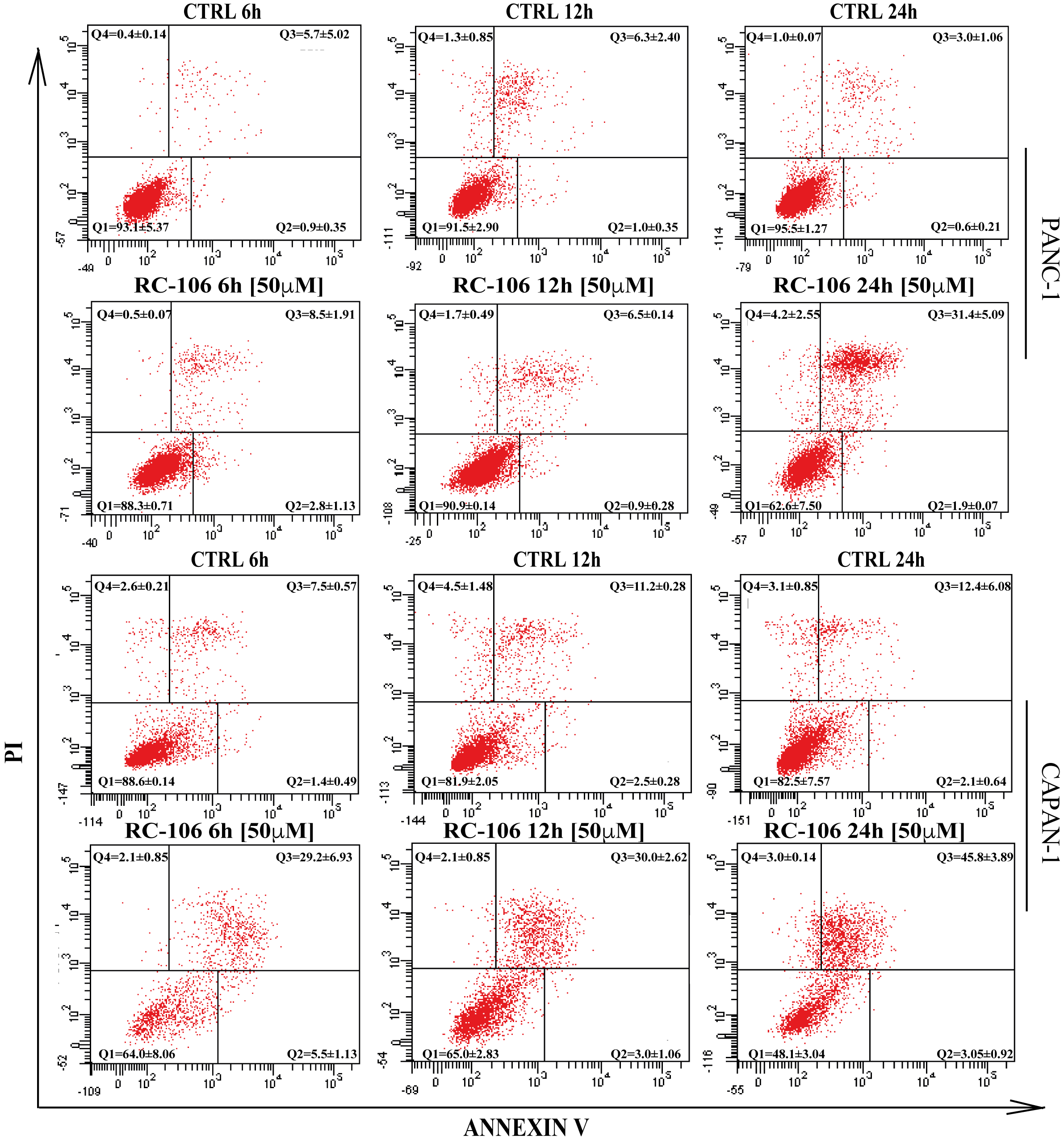

Supplement: Supplementary file 1 [file ijms-21-09012-s001.zip › Supplementary_figure2.tif]

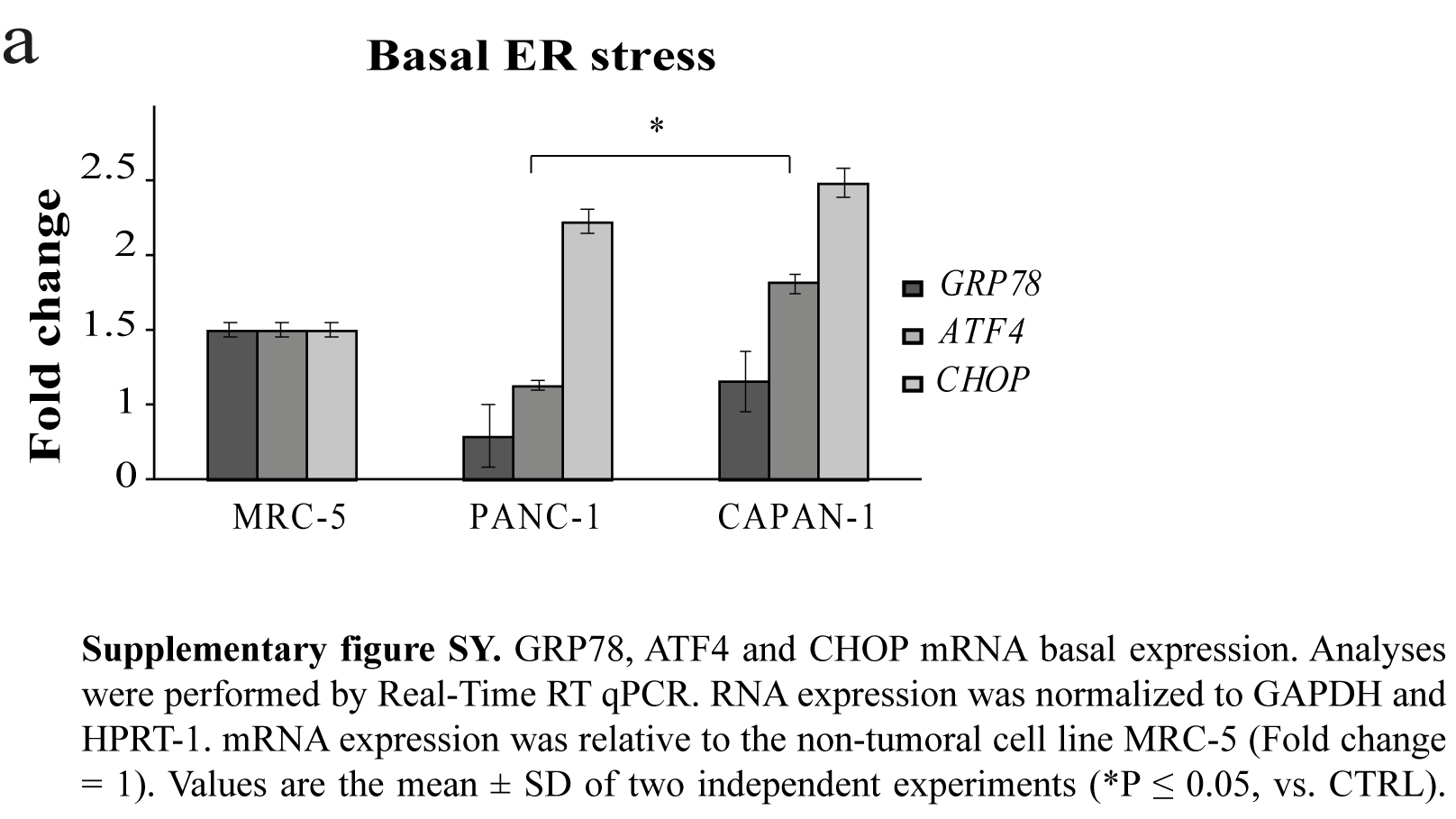

Supplement: Supplementary file 1 [file ijms-21-09012-s001.zip › Supplementary figure 3.tif]

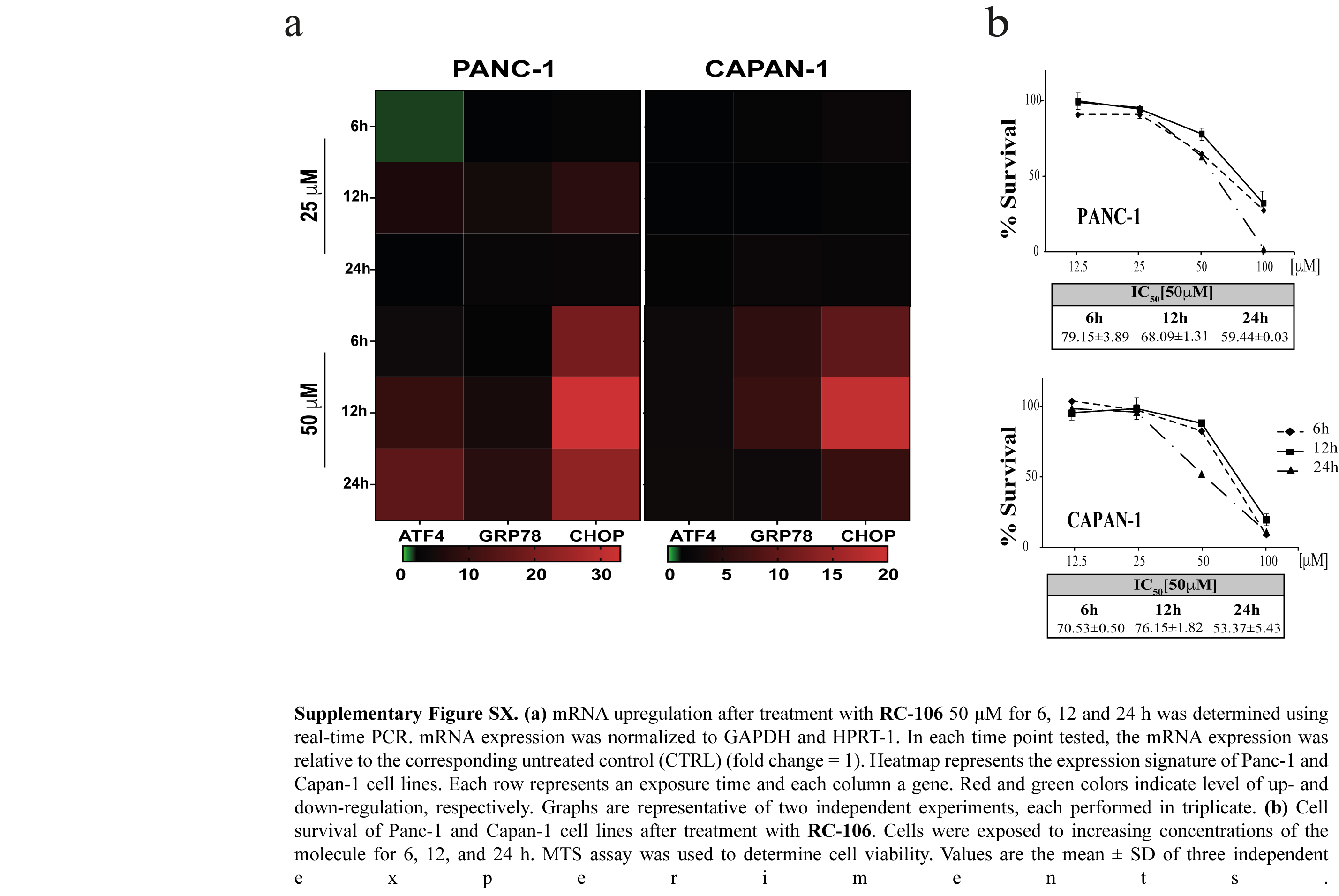

Supplement: Supplementary file 1 [file ijms-21-09012-s001.zip › Supplementary Figure 1.tif]

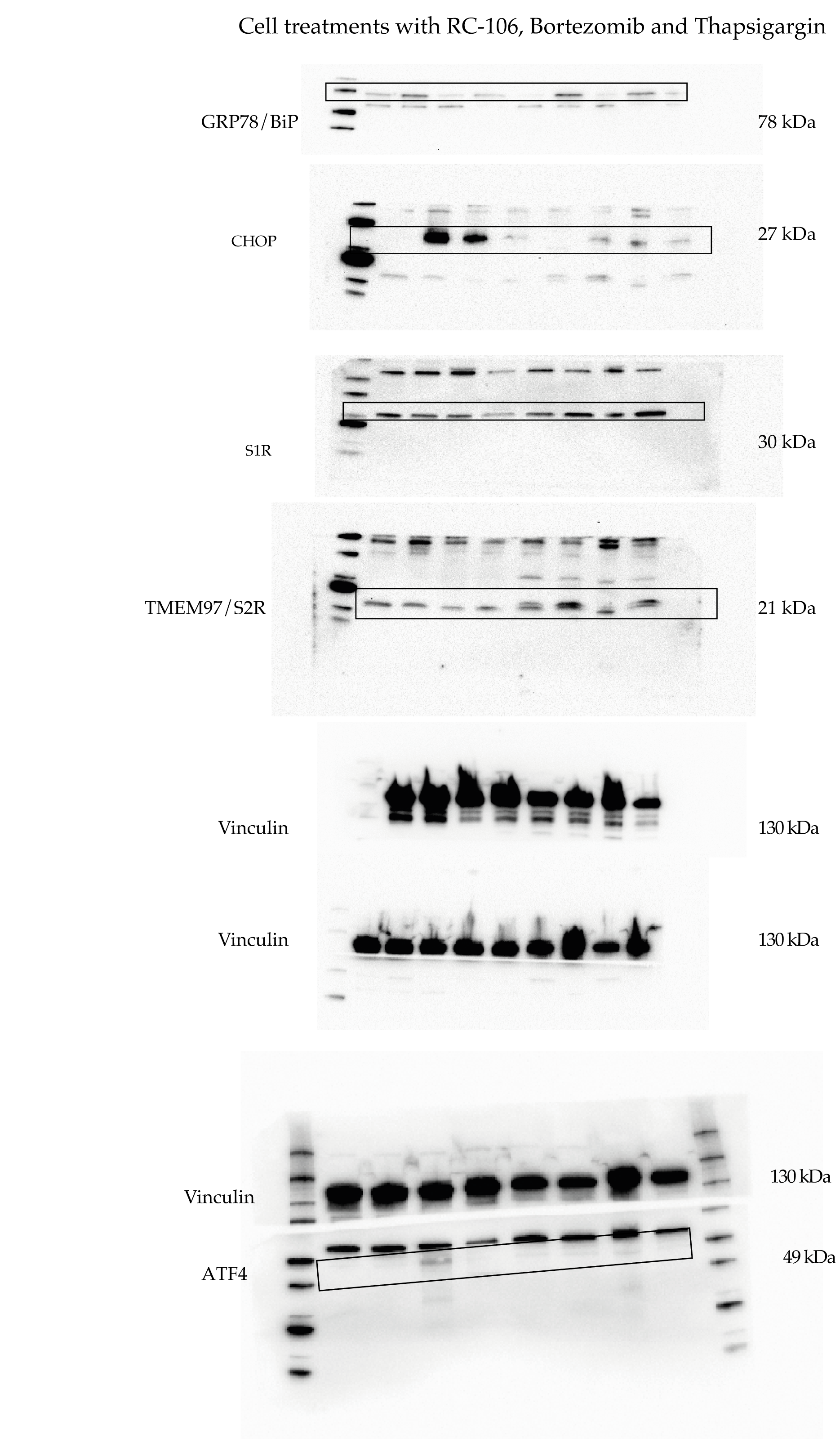

Supplement: Supplementary file 1 [file ijms-21-09012-s001.zip › Supplementary_figure 4.tif]

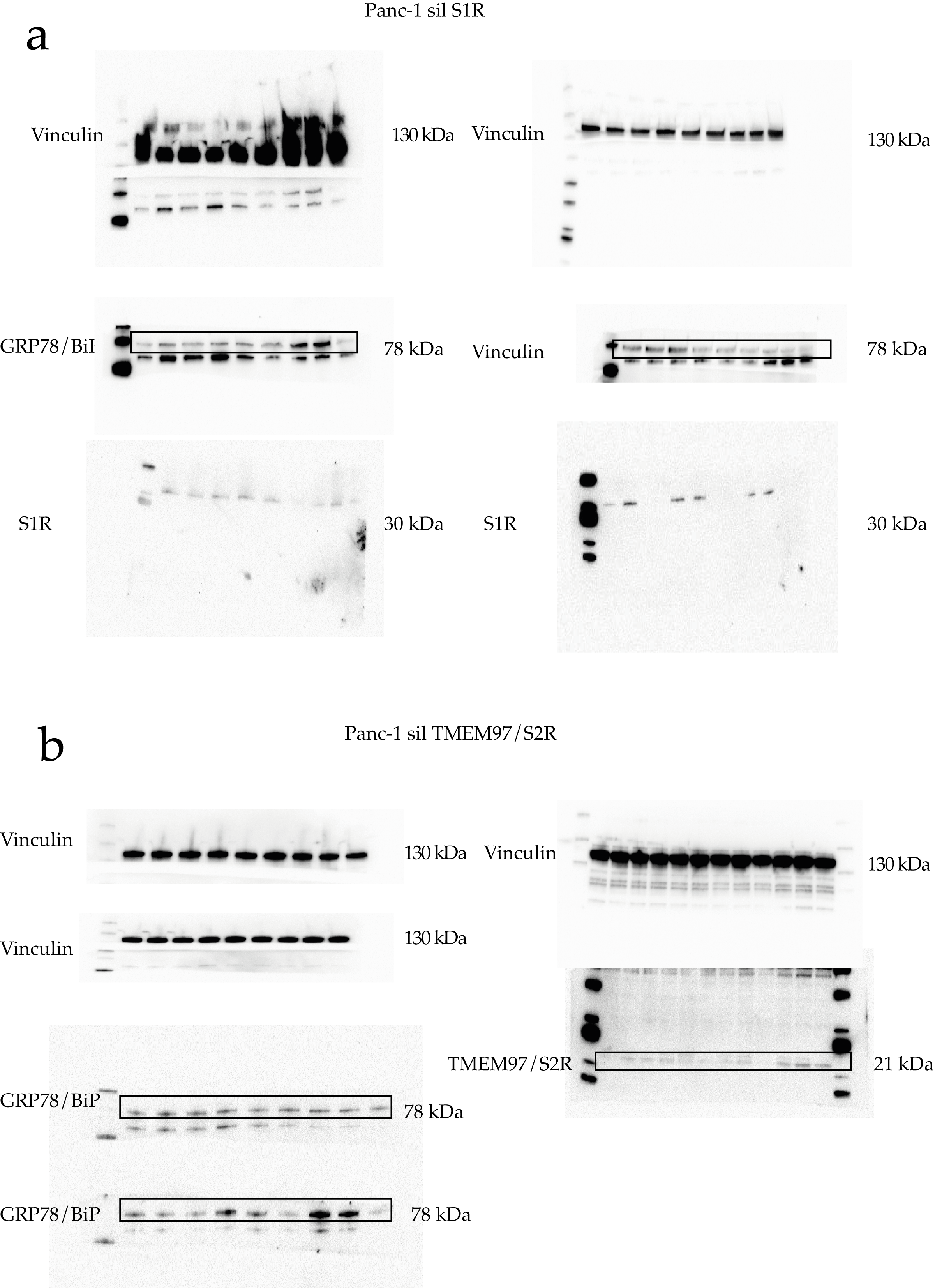

Supplement: Supplementary file 1 [file ijms-21-09012-s001.zip › Supplementary_figure5.tif]

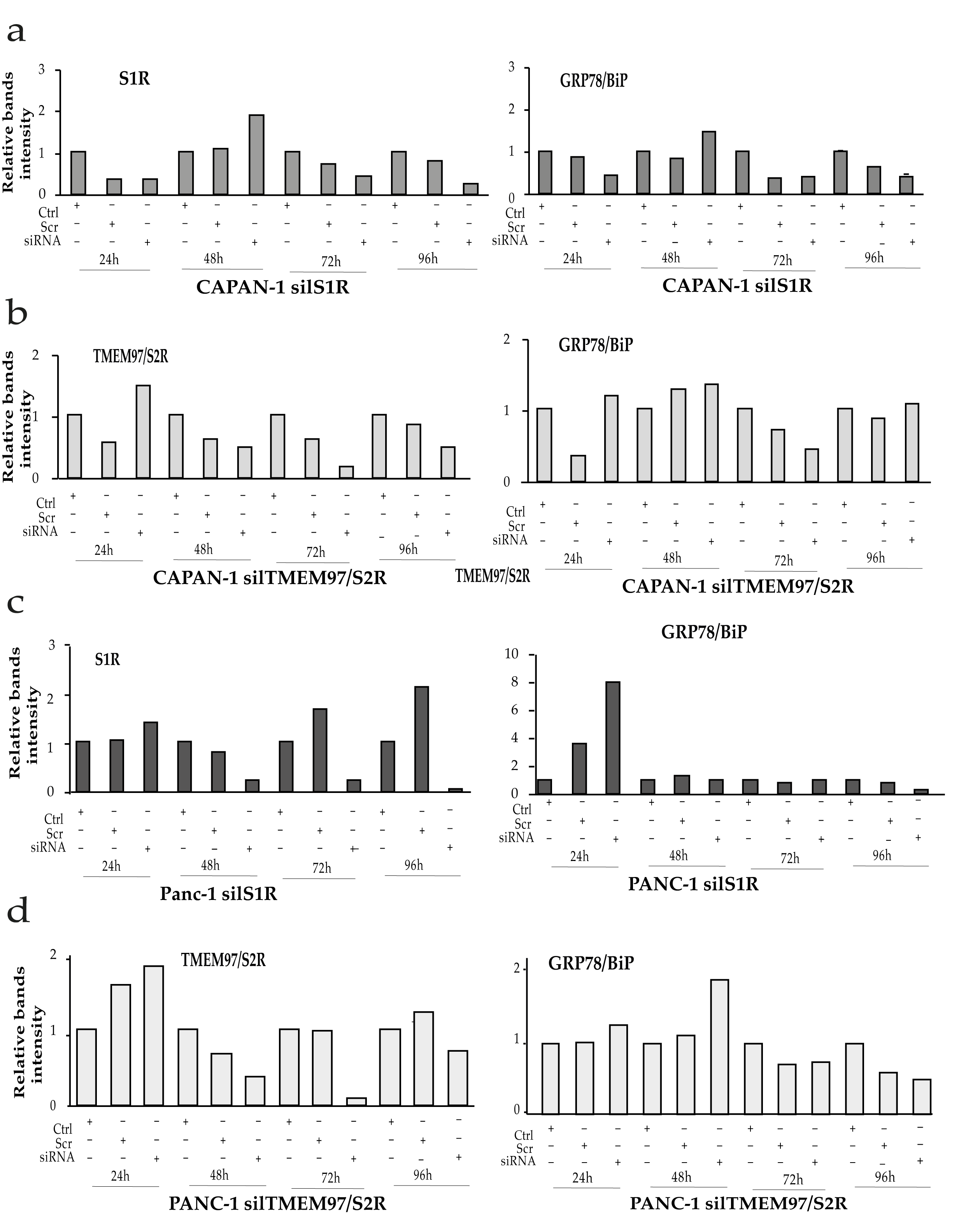

Supplement: Supplementary file 1 [file ijms-21-09012-s001.zip › Supplementary_figure7.tif]

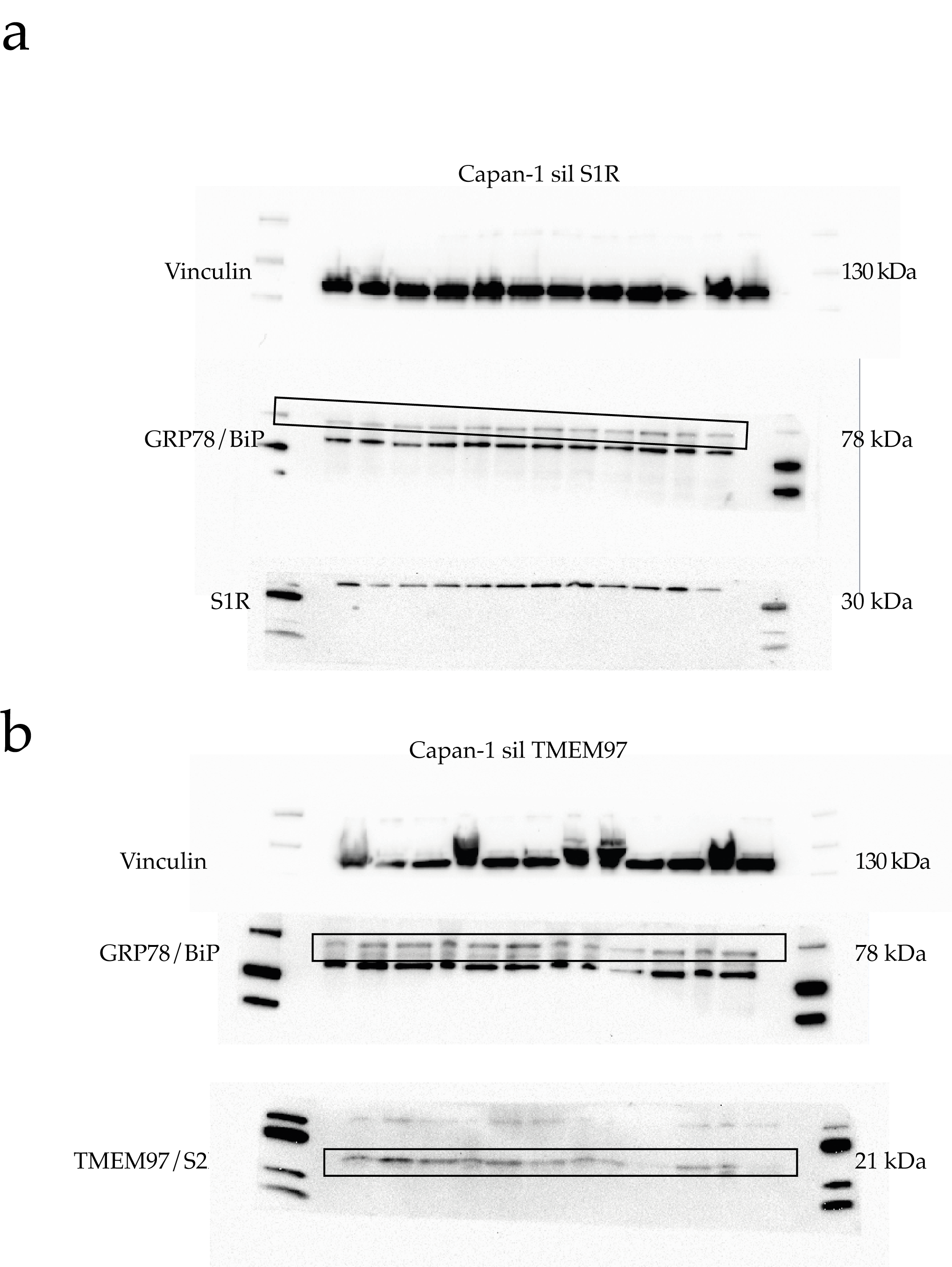

Supplement: Supplementary file 1 [file ijms-21-09012-s001.zip › supplementary_figure6.tif]
